# Disparities in COPD Hospitalizations: A Spatial Analysis of Proximity to Toxics Release Inventory Facilities in Illinois

**DOI:** 10.3390/ijerph182413128

**Published:** 2021-12-13

**Authors:** Stacey Brown-Amilian, Yussuf Akolade

**Affiliations:** Department of Geography and GIS, Southern Illinois University, Edwardsville, IL 62026-1459, USA; yussufolatunjiakolade@gmail.com

**Keywords:** medical geography, TRI facilities, COPD, hospital discharge data

## Abstract

Disproportionate distribution of air pollution is a major burden on the health of people living in proximity to toxic facilities. There are over 1000 Toxics Release Inventory (TRI) facilities distributed across the state of Illinois. This study investigates and spatially analyzes the relationship between chronic obstructive pulmonary disease (COPD) hospitalizations and toxic emissions from TRI facilities. In addition, this study investigates the connection between COPD hospitalizations and socioeconomic variables. Accounting for dispersion of air pollution beyond the TRI facilities source was attained using the inverse distance weighting interpolation approach. Multiple statistical methods were used including principal components analysis, linear regression, and bivariate local indicators of spatial association (BiLISA). The results from the linear regression model and BiLISA clustering maps show there is a strong connection between COPD hospitalizations and socioeconomic status along with race. TRI emissions were not statistically significant, but there are three major clusters of high COPD hospitalizations with high TRI emissions. Rural areas also seem to carry a higher burden of pollution-emitting facilities and respiratory hospitalizations.

## 1. Introduction

The influence of the built environment on health has been a topic of research for many years [1,2,3]. Past and current research has revealed the negative influence of poor air quality on health, especially regarding respiratory effects [4,5,6]. In addition, researchers have documented the impacts of air pollution across the sociodemographic spectrum and how these impacts affect low-income and minority populations at a higher level than their counterparts [7,8]. Substances released from toxic facilities pose a major threat to the environment and to public health, especially to people living in close proximity [5,9,10].

A program to track, record, and manage toxic chemicals emitted by industrial facilities, known as the Toxics Release Inventory (TRI) program, was created in the U.S. in 1986. As a result, 187 air pollutants were declared harmful to the environment and public health by the U.S. Environmental Protection Agency (EPA), and many of these are suspected of causing serious health effects [11]. Prior to the creation of the TRI program, the environmental movement in the 1950s and 1960s presumed that pollution and environmental degradation impacted people’s health and the environment equally. However, researchers in the 1980s and 1990s established that this was incorrect and found differences in who is being affected, especially in regards to the siting of hazardous waste locations [12]. Subsequently, researchers in the United States have focused more on the unequal geographic distribution of environmental health hazards, the potential disproportionate effects to minorities and low-income communities, and, therefore, differential exposure risk [5].

The study of environmental impacts of pollution is a broad spectrum that can be assessed through the lenses of health disparities or environmental justice, or both. Findings have shown that chronic obstructive pulmonary disease (COPD), diabetes, myocardial infarction, and congestive heart failure occur at greater risk with air pollution [13]. In addition, the disproportionate distribution of pollution sources shown in various studies highlights a higher concentration of pollution hazards in minority communities, which is a form of environmental injustice [7,14]. Therefore, it is difficult to only focus on proximity without also focusing on demographics and public health [9].

Exposure to air pollution is categorized as one of the leading public health concerns and causes of mortality and morbidity [15]. The association of daily morbidity and mortality to poor air quality is mostly linked to vulnerable people with pre-existing cardiorespiratory disease, including COPD, and the elderly [4,6]. An association was found between COPD mortality and pollution from particulate matter in southeast China [16]. Thus, there is a need to examine the impacts of outdoor air pollution on the residents living near TRI facilities along with hospitalizations for respiratory diseases, such as COPD. The main purpose of this study was to investigate and spatially analyze how the environmental impacts of proximity to TRI facilities could influence COPD hospitalizations in Illinois. The secondary purpose of this study was to understand what demographic characteristics affect COPD hospitalizations.

Illinois has previously been studied for pollution and these studies have established the high rate of pollution in the Metro East of Illinois [9,14,17], where Granite City steel mills account for 14 percent of the region’s pollution with 52 different air toxins. Second-ranked Sauget’s Solutia chemical plant released 46 different air toxins, and the third-ranked was Wood Rivers’ Tosco refinery, with 50 different air toxins [14]. It was reported that four of the contiguous Sauget, IL facilities cumulatively produce one-fifth of the entire pollution in the St. Louis region, and they are located in proximity to the region’s most vulnerable community, East St. Louis, of which 98% were African American, 48.8% lived below the poverty level, and 25% were unemployed in the 1990 census [14]. A more recent study using TRI facilities, roadways, industrial land uses, respiratory hospitalizations, and demographics found that poverty was the main driver for hospitalizations rather than pollution-generating facilities, roads, or land uses [9].

This study contributes to the growing body of literature focused on pollution, health, and demographics along with Illinois studies by focusing on COPD hospitalization and proximity to TRI facilities across the entire state of Illinois.

## 2. Materials and Methods

### 2.1. Description of Data

Data needed for this project came from three sources: the EPA TRI, Illinois Department of Public Health (IDPH), and the United States Census Bureau. All data were downloaded for the year 2011. Multiple processes were applied in this study to provide both statistical and geospatial results. ArcMap 10.7, ArcGIS Pro 2.8, and GeoDa 1.20 were used for geospatial analysis and Statistical Package for Social Science (IBM SPSS 28.0) was used for the statistical analysis.

**Figure 1:**
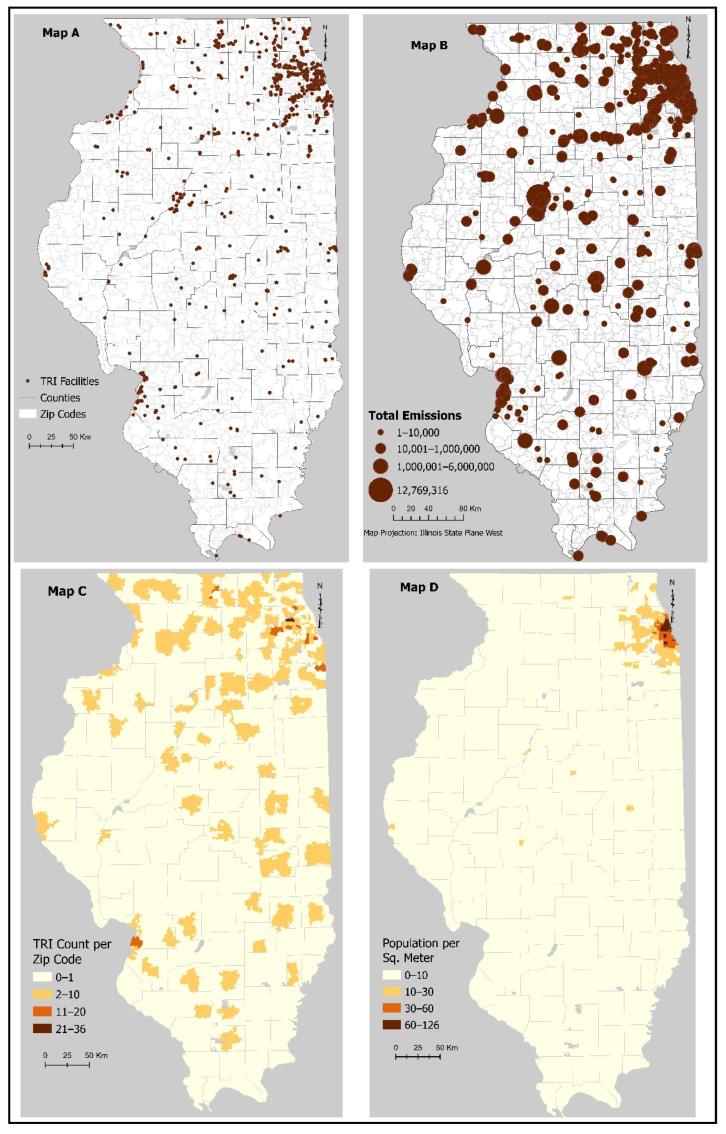
(**A**) TRI Counts, (**B**) Emissions, and (**C**) Counts per Zip Code along with (**D**) Population Density for Illinois in 2011.

The EPA TRI facilitates the reporting of toxic releases in the United States and allows users to generate reports on releases, transfers, and waste management. TRI facilities (1083) were found in almost all the zip codes in Illinois. However, only the TRI facilities (847) with releases more than 1 lb were analyzed and are shown in Figure 1, Maps A and B. The volume of toxic emissions differs by zip code and varies by facility. A preliminary analysis showing the distribution of TRI facilities in Illinois shows that zip codes in Cook County have the largest number of toxic release facilities. Some zip codes have TRI facilities with high emissions, while some have little to no emissions, as seen in Figure 1, Map B. Figure 1, Map C reveals a higher amount of releases in the more rural areas of the state compared to the urban areas. Population per zip code is shown in Figure 1, Map D.

Using ArcMap 10.7, the total amount of toxic releases per facility was interpolated using IDW to capture the extent of local surface variations. The first step of the process was to convert the TRI facilities and the TRI emissions into raster layers by using the convert to raster function. This step produced a pollution surface for the entire state of Illinois. Next, this raster layer was interpolated using the IDW function. Third, the interpolated surface was converted into a point shapefile by the convert to vector function. This point layer contained the interpolated TRI emissions. Finally, a spatial join combined the TRI point layer to each Illinois zip code. This procedure transferred vectorized TRI data into zip code polygons as well as provided the mean (average) of the vectorized TRI emissions for each zip code.

The IDPH provides inpatient and outpatient data collected by its division of patient safety and quality on discharge data from all Illinois specialty hospitals, acute care hospitals, and emergency hospitals. IRB approval was waived as the data contains no identifiable information for Illinois residents. This study includes all patients presenting or admitted for COPD (ICD-9 codes 492–496) during 1 January 2009–31 December 2011. Information obtained from the discharge records included a reason for utilizing the inpatient or emergency department, such as diagnosis, admission date, cost, and a unique hospital identifier, as well as information related to the patient, such as age, ZIP code of residence, race/ethnicity, and gender [18]. Age-standardized hospitalization rates were computed using the direct method to the projected year 2000 US population. Hospitalizations were organized by zip code and by 5-year age increments. The number of hospitalizations per age group was divided by the appropriate age population per zip code, multiplied by 100,000 and then multiplied by the corresponding age distribution value. Each age group was used during standardization, from infants to one patient aged 109.

The United States Census Bureau is the source that provides different kinds of data related to geography and demographics. The shapefile of Illinois’ zip codes can be found on the U.S. Census Bureau’s website. The Tiger/Line shapefiles and demographic data for all zip codes in Illinois were extracted from the U.S. Census Bureau through the American Community Survey (ACS) five-year estimate for 2011, using Table S0601 [19].

### 2.2. Principal Components Analysis

There are 11 independent variables listed in Table 1. Each of these variables are important to understanding COPD hospitalizations. However, due to multicollinearity issues, an additional procedure was performed to remove the correlation between the variables [20]. PCA was performed on the independent variables, and five components were extracted using the equamax rotation, which explained 80.1% of the variance. Table 2 describes the new factors used in the statistical analysis.

### 2.3. Statistical Analysis Using Linear Regression

A linear regression model was utilized with the age-standardized COPD hospitalization rate as the dependent variable and the five components from the PCA as the independent variables. Residuals and ANOVA results were checked for accuracy.

### 2.4. Geospatial Analysis in ArcGIS and GeoDa

Spatial patterns are uncovered by analyzing the spatial distribution of the study’s dependent and independent variables. These maps enable us to interpret the zip codes with the highest, intermediate, and least emissions, demographics, and hospitalizations. Local Indicators of Spatial Association (LISA) are a geostatistical tool that gives an indication of the extent of significant spatial clustering of similar values around a specific observation. LISA is used to identify local spatial clusters, known as hot spots or cold spots. These clusters can be used as the basis for a test on the null hypothesis of no local spatial association [21]. While it is important to understand how the data clusters over the study area, the clusters do not tell us where in space those clusters occur. Getis-Ord G statistic allows us to confirm that there are clusters occurring in our data, but not where. Getis-Ord G* or the local Moran’s I reveals where in the study area clustering is occurring and between certain variables. Therefore, it is imperative to know that there is clustering occurring with the G statistic along with the location of these clusters, which is achieved by using the bivariate cluster maps from GeoDa.

## 3. Results

### 3.1. Descriptive Results

There were approximately 293,000 hospitalization cases in 1321 zip codes for COPD. The hospitalization dataset indicates that the youngest population (under 5) is the most affected, with an average age of 33 for the hospitalizations due to COPD. There are 847 pollution sources with more than one pound of toxic release in the study area. Table 3 shows the top five zip codes with the highest toxic releases in 2011. Peoria County is the only county on the list twice with two zip codes with the highest amount of toxic releases, which amounts to over 16% of the pollution for the state of Illinois.

**Figure 2:**
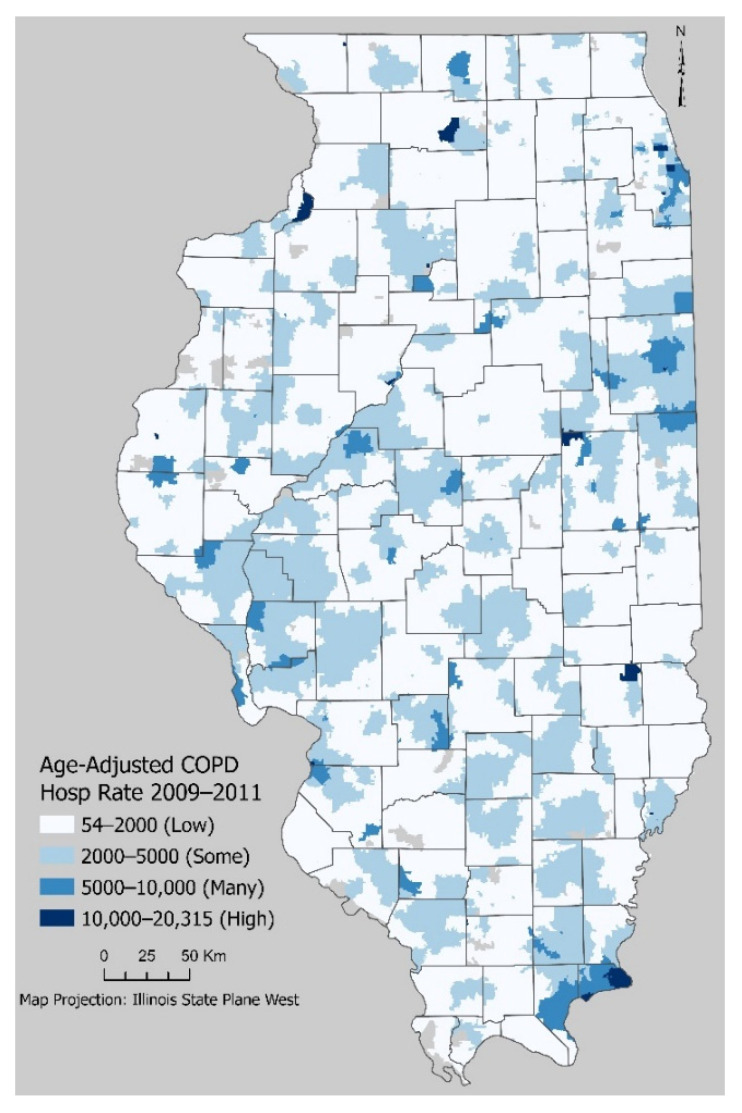
COPD Age-Standardized Hospitalization Rate per 100,000.

Figure 2 shows the COPD hospitalization age-standardized rate per 100,000 persons, which displays four areas with relatively high hospitalizations along with six zip codes that have extremely high hospitalization rates. Hospitalization records only show extreme cases and therefore, do not reflect patients that have COPD managed with medication. COPD hospitalizations do not seem to have a clear-cut geographical pattern, with metropolitan areas and rural areas showing high hospitalization rates.

### 3.2. Linear Regression Results

Results from the linear regression model reveal that there does not seem to be a statistically significant relationship between pollution and COPD hospitalizations or the Hispanic and population density factor (Table 4). Low SES and the race and jobs factors are significant at the 0.001 level and the male factor is significant at the 0.1 level. The most significant results are focused on factors related to low income and race between Whites and African Americans.

### 3.3. Bivariate Cluster Maps—Local Indicators of Spatial Autocorrelation

While the previous results show the influence of the variables across the study area, the focus now shifts to how neighboring values influence the dependent and independent variables, simultaneously. The spatial association between the components and COPD hospitalization was established using BiLISA to present clustering maps for each of the five factors and COPD hospitalization.

**Figure 3:**
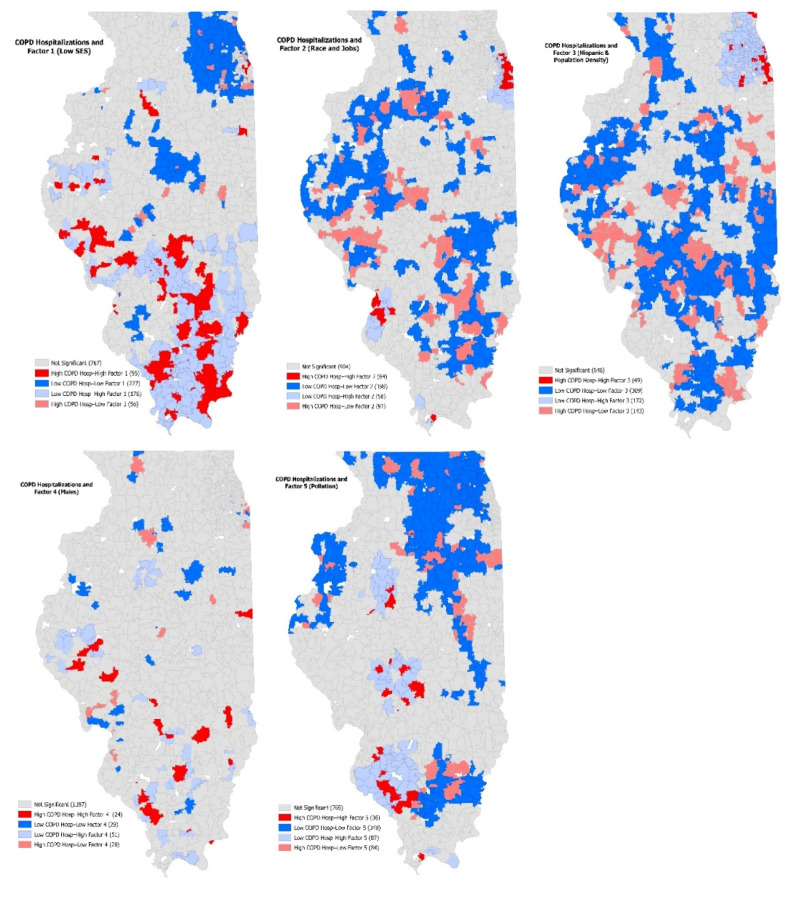
Bivariate LISA maps for each component and age-adjusted COPD hospitalization rate.

The BiLISA legend depicts the combination of the COPD hospitalization (first) and the component involved (second) ranking the influence of neighboring values as well as the number of zip codes in each category. The high-high category details that neighboring values for the two variables are high. Figure 3 shows the COPD hospitalization rate and the five components as bivariate LISA cluster maps. Map A presents the hospitalizations and factor 1 (low SES). Ninety-five zip codes show high COPD hospitalizations with a high percentage of low SES zip codes. Rather than seeing clustering in the major metropolitan areas, more high-high clusters are found in the southern portion of the state and along the Mississippi River. The majority of low-low zones exist in the northern Chicago suburbs and near Springfield, Illinois in the central part of the state.

Figure 3, Map B presents the COPD hospitalization rate and factor 2, which is African Americans, unemployment, and Whites, with a negative result. This factor represents the segregation of the two races in the state with the high-high clusters existing in Chicago and in East St. Louis. This result reveals that African Americans tend to reside in the major metropolitan areas of the state. The high-low clusters represent high COPD hospitalizations and low race and jobs. Again, these results show more impact in the rural areas and away from the major metropolitan areas of the state.

Figure 3, Map C shows the COPD hospitalization rate and factor 3, which represents Hispanics and population density. Only one area of the state is shown as high-high clustering, which exists in the southern portion of the Chicago metro area. The high-low clustering represents high COPD hospitalizations and low Hispanic/population density. The majority of these clusters are found in high-high clusters in Map A. Map D presents the COPD hospitalization rate and the percent male factor. Very few zip codes are shown in the map as having any significant clustering, but again, the southern portion of the state shows high-high clustering.

Finally, Map E shows the COPD hospitalizations and pollution component. Three major groups of zip codes appear within the high-high clusters, which represent small metropolitan areas: Peoria to the north, Springfield in the center, and the Metro East in the south. Some of the major polluting facilities are located near these small metro areas as shown in Table 3. Additionally, there are large groups of zip codes with both low COPD hospitalizations and low pollution levels.

## 4. Discussion

This study found a statistically significant relationship between low socioeconomic status, race and unemployment, and COPD hospitalizations. Financial variables, such as median income and percentage of the population in poverty, population without medical insurance, and low educational attainment, were also statistically significant with COPD hospitalizations.

The results of this study yield similar results to other research related to low-income residents having a higher burden of poor health [7]. Additionally, the results indicated that African Americans are more likely to be hospitalized due to COPD than their White counterparts, but Hispanics do not seem to have any additional hospitalizations. However, the statistical results do not find that higher pollution relates to higher COPD hospitalizations.

When analyzing the location of the TRI facilities, urban areas do have a higher number of these facilities, yet their emissions are low. The rural areas of the state have fewer numbers of facilities yet have the highest emissions from TRI facilities. When examining the COPD hospitalizations, the areas of the state with the highest hospitalizations exist in the rural areas. Analyzing the BiLISA maps (Figure 3), there are four clusters that have both high COPD hospitalizations and high TRI emissions, and these are all found outside of major metropolitan areas. Therefore, this study adds to the literature to include rural areas or non-metropolitan areas for a broader picture of health effects of pollution [22].

Four major limitations existed. The use of zip codes is not an ideal geographic unit for evaluation as these are utilized for efficient mail delivery routes, rather than demographic analysis. Even though this is not an ideal geographical unit to study, all data were analyzed at the zip code level and the health data is only available at this level. The second limitation was the use of data from 2011. While the data is almost 10 years old, it does provide a snapshot of not only hospitalizations for the area, but also what is occurring with TRI facilities at that time. The availability of most of the demographic data for this time provides a solid overview of the demographics for this year. Ideally, data that are more recent would be used, but the ability for all three datasets to maintain the same year is a strength.

An additional limitation encountered is the unavailability of health insurance data for 2011. The U.S. Census Bureau started adding health insurance data in 2012, and this data was used with the other socioeconomic characteristics for 2011. A final limitation is regarding the hospitalization data. This data represents only those patients that go to a hospital for care. This health data does not record the data of people who have the disease under control through consultation and medications. Therefore, only the extreme cases of COPD are presented with the hospitalization results. COPD can be effectively managed under a doctor’s care and these numbers do not reflect the true number of Illinois residents dealing with COPD.

There is a need to perform this study with more recent COPD hospitalizations and TRI data along with an examination of how the toxic compound makeup of the emissions impacts respiratory health. Incorporating additional years can identify changes regarding hospitalizations and pollution along with monitoring the zip codes with high categories of both respiratory issues and emissions. Adding this longitudinal component could show how reducing the reliance on fossil fuels could also reduce health burdens.

## 5. Conclusions

This study’s findings contribute to the literature of the impact of pollution on health with its focus on every zip code in one state. Respiratory health in the form of COPD hospitalizations is statistically significant to components that include low socioeconomic status, race, and unemployment as well as percent male. These results also reveal that many of the TRI emissions impact more rural areas of the state. Therefore, it is imperative to include rural areas and not only metropolitan locations when studying the health impacts of pollution. While the two metropolitan areas of the state, Chicago and Metro East St. Louis, did have significant influences, the most clustering occurred in the rural, central, and downstate areas of Illinois. A specific area to focus on would be southwest Illinois. In addition, this study addressed and accounted for the dispersion of pollution by assigning TRI emissions to every zip code via interpolation. Policymakers can utilize this information for decision-making in helping to keep a safer and healthy environment for every Illinois resident across all socioeconomic backgrounds and in both metropolitan and non-metropolitan areas.

## Figures and Tables

**Table 1:** Independent variables.

Variable	Description
Pollution *	Average amount of release from TRI facilities (gained from interpolation) Impacts of TRI release on the population of every zip code
Population density	Total population/Land Area
Population male	Percentage of male population
White	Percentage of the White population
Black	Percentage of the Black population
Hispanic	Percentage of the Hispanic population
Less than high school	Percentage of people without a high school diploma
Unemployed	Percentage of people unemployed
Income	Median Income of every zip code in dollars
Poverty	Percentage of the population in extreme and moderated poverty
Medically Uninsured **	Percentage of people without medical/health insurance

* Data source is EPA TRI Website; ** Data source Table S2701 ACS Census.

**Table 2:** Results from PCA and new variables name.

Factor	Components	New Variable Name
1	Less than High School, Median Income (negative), Poverty and Medically Uninsured	Low SES
2	White (negative), Black, Unemployed	Race and Jobs
3	Hispanic and Population Density	Hispanic and Density
4	Male	Male
5	Pollution	Pollution

**Table 3:** Zip Codes with the greatest amount of toxic releases.

Zip Code	County	TRI Count	Total Volume	Avg. Volume	% of Total Toxic Pollution
62257	Washington	1	19,589,794	19,589,794	18.4
61604	Peoria	3	10,798,242	3,599,414	10.1
60421	Will	2	7,029,355	3,514,678	6.6
61607	Peoria	4	6,588,564	1,647,141	6.2
61081	Whiteside	4	5,635,071	1,408,768	5.3

**Table 4:** Linear Regression model for rate of COPD hospitalizations.

Variable	Un Standardized B	Std. Error	Beta	T	Sig.	95% Confidence Interval
Constant	2204.13	51.67		42.637	<0.001	2102.72–2305.54
Factor 1	696.46	51.71	0.316	13.467	<0.001	595.01–797.91
Factor 2	908.86	51.71	0.413	17.575	<0.001	807.41–1010.31
Factor 3	9.92	51.71	0.005	0.192	0.848	−91.53–111.37
Factor 4	−133.58	51.71	−0.061	−2.583	0.010	−235.04–−32.14
Factor 5	44.61	51.71	0.020	0.863	0.389	−56.94–146.06

## Data Availability

Not applicable.
